# Pharmacology and Therapeutic Potential of Finerenone: A Novel Third-Generation Nonsteroidal Mineralocorticoid Receptor Antagonist

**DOI:** 10.7759/cureus.87706

**Published:** 2025-07-11

**Authors:** Mahmoud M Ramadan, Shaha Alajmi, Jude Aldouseri, Manar Alajmi, Eva A Tello, Abdul-Majeed O Mahdi, Ahmad J Aladwani, Abdulrahman E Alayyaf, Ousama Mahdi, Mohammed Elmahal

**Affiliations:** 1 Department of Cardiology, Faculty of Medicine, Mansoura University, Mansoura, EGY; 2 Department of Clinical Sciences, College of Medicine, University of Sharjah, Sharjah, ARE; 3 Department of Cardiology, University Hospital Sharjah, Sharjah, ARE; 4 Department of Diabetes and Endocrinology, Khartoum North Teaching Hospital, Khartoum, SDN

**Keywords:** cardiovascular benefit, chronic kidney disease, chronic kidney disease (ckd), finerenone, heart failure, mineralocorticoid receptor antagonist, reduced ejection fraction, serum potassium level, third generation

## Abstract

Finerenone is a novel third-generation nonsteroidal mineralocorticoid receptor antagonist (MRA) that has demonstrated efficacy in treating chronic kidney disease (CKD) and heart failure with reduced ejection fraction (HFrEF). Unlike traditional steroidal MRAs such as Spironolactone and Eplerenone, Finerenone offers high selectivity for MR, resulting in reduced off-target effects, including hyperkalemia and antiandrogenic side effects. It exerts renoprotective and cardioprotective effects through its anti-inflammatory and antifibrotic actions, making it a good therapeutic option for patients with CKD and HFrEF. The pharmacokinetic profile of Finerenone demonstrates rapid absorption, satisfactory oral bioavailability, and predictable systemic clearance, contributing to its efficacy and tolerability. Clinical trials, including advanced Phase III studies, have established Finerenone’s ability to delay CKD progression, reduce proteinuria, and improve cardiovascular (CV) outcomes (such as hospitalization for heart failure and CV death) in patients with diabetes mellitus and CKD. Compared to other MRAs, Finerenone demonstrates improved tolerability; however, clinically significant risks such as hyperkalemia necessitate proactive monitoring and protocol-driven management. Additionally, its unique molecular structure and mechanism of action make it a safer alternative for long-term use in diverse populations. While initial results are promising, further research is necessary to evaluate the long-term outcomes and explore its full therapeutic potential, including novel applications in high-risk patients and biomarker-driven treatments. These findings position finerenone as a valuable addition to current therapies for CKD and HFrEF, though broader adoption awaits real-world validation of its safety and cost-effectiveness.

## Introduction and background

Chronic kidney disease (CKD) and heart failure (HF) represent intertwined global health challenges, collectively affecting over 850 million people worldwide and driving substantial morbidity, mortality, and healthcare costs [1,2]. Central to their pathophysiology is the overactivation of the mineralocorticoid receptor (MR), a key mediator of aldosterone-induced sodium retention, potassium excretion, fibrosis, and inflammation [3,4]. Mineralocorticoid receptor antagonists (MRAs) counteract these effects, offering renal and cardiovascular (CV) protection. However, traditional steroidal MRAs, such as Spironolactone and Eplerenone, are limited by nonselective receptor binding, leading to dose-dependent hyperkalemia (19%-24% incidence in CKD) and endocrine side effects (e.g., gynecomastia in 10%-15% of patients) that restrict their use in high-risk populations [5-7].

The rising prevalence of cardiorenal diseases, fuelled by aging populations and epidemics of type 2 diabetes mellitus (T2DM) and hypertension, has intensified the need for MRAs that balance efficacy with safety [1,2]. Steroidal MRAs, while foundational, often necessitate discontinuation or dose reduction in advanced CKD due to hyperkalemia risk, leaving many patients undertreated [5,7]. Finerenone, a third-generation nonsteroidal MRA, emerged from targeted drug design to address these limitations. In vitro studies demonstrated 800-fold higher MR selectivity over androgen and progesterone receptors compared to Spironolactone, minimizing off-target hormonal interactions [8,9]. Preclinical models further revealed balanced tissue distribution, favoring cardiac and renal MR blockade while reducing renal tubular potassium retention, a critical advance for CKD populations [9,10]. These mechanistic advantages translated clinically: phase 3 trials (FIDELIO-DKD, FIGARO-DKD) demonstrated finerenone’s ability to reduce the risk of kidney failure by 18% (hazard ratio, HR, 0.82; 95% confidence interval, CI, 0.73-0.93) and CV events by 14% (HR, 0.86; 95% CI, 0.75-0.99) in patients with T2DM and CKD, with hyperkalemia rates of 14% vs. 19%-24% for Spironolactone [7,11,12].

This review examines the pharmacology, clinical efficacy, and safety profile of Finerenone, contextualizing its role within the evolving landscape of MR antagonism. It emphasizes finerenone’s potential to address unmet therapeutic needs in CKD and HF, while also acknowledging ongoing challenges, such as the risk of hyperkalemia in advanced CKD and the need for long-term mortality data.

## Review

Evolution of MRAs: from spironolactone to finerenone

The evolution of MRAs has been driven by a deepening understanding of aldosterone’s pathophysiological role and the need to address limitations of prior therapies. Aldosterone, a key effector of the renin-angiotensin-aldosterone system (RAAS), promotes sodium retention, potassium excretion, and vascular remodeling, contributing to hypertension, fibrosis, and end-organ damage in CKD and HF [3,4]. Early recognition of aldosterone’s harmful effects, such as its role in cardiac fibrosis and renal inflammation, established MR antagonism as a therapeutic target [13,14]. However, translating this knowledge into safe and effective therapies required iterative advancements in receptor selectivity and pharmacokinetics.

First-Generation MRA: Spironolactone

Discovered in the 1960s, Spironolactone became the first clinically viable MRA. While it effectively blocked aldosterone’s effects, its steroidal structure conferred nonselective binding to androgen and progesterone receptors [5]. This off-target activity caused dose-limiting endocrine side effects, including gynecomastia (10%-15% of male patients) and menstrual irregularities, which restricted its long-term use [5,6]. Despite these drawbacks, Spironolactone demonstrated mortality benefits in severe HF (Randomized Aldactone Evaluation Study, RALES, trial, 30% reduction [6]), validating MR blockade as a strategy. However, its nonselectivity and hyperkalemia risk in CKD underscored the need for improved agents.

Second-Generation MRA: Eplerenone

Eplerenone, developed in the 2000s, retained a steroidal backbone but exhibited higher MR selectivity, minimizing sex hormone receptor binding [15]. This reduced endocrine toxicity, enabling broader use in postmyocardial infarction HF (Eplerenone Post-Acute Myocardial Infarction Heart Failure Efficacy and Survival Study, EPHESUS, trial: 15% mortality reduction [15]). However, its steroidal nature still predisposed patients to hyperkalemia, particularly in renal impairment, due to lipophilicity-driven renal tubular accumulation [7,11]. These limitations highlighted the need for nonsteroidal MRAs with optimized tissue distribution.

Third-Generation MRA: Finerenone

Finerenone, a 2010s innovation, was designed to overcome steroidal shortcomings. Its dibenzothiophene-based, nonsteroidal structure eliminated sex hormone receptor binding, resolving endocrine side effects [5,9]. Preclinical studies revealed a balanced tissue distribution, preferentially targeting cardiac and renal MRs, which reduced renal potassium retention and the risk of hyperkalemia compared to steroidal MRAs [9,10]. Phase 3 trials (Finerenone in Reducing Kidney Failure and Disease Progression in Diabetic Kidney Disease, FIDELIO-DKD, and Finerenone in Reducing Cardiovascular Mortality and Morbidity in Diabetic Kidney Disease, FIGARO-DKD) confirmed its cardiorenal benefits in T2DM and CKD, with hyperkalemia rates comparable to placebo when managed proactively [11,12]. This evolution reflects a direct response to prior generations: structural refinement to enhance selectivity, mitigate toxicity, and align pharmacokinetics with pathophysiological targets.

Each MRA generation emerged from lessons learned: Spironolactone’s efficacy established MR antagonism as vital, Eplerenone’s selectivity addressed hormonal toxicity, and Finerenone’s nonsteroidal design optimized safety for chronic use in high-risk populations. This progression underscores how translational insights into aldosterone’s mechanisms directly informed therapeutic innovation.

Finerenone mechanism of action and pharmacokinetics

Mechanistic Rationale

Finerenone is a potent, orally available, nonsteroidal MRA that selectively blocks aldosterone and cortisol binding to MR without modulating 11β-hydroxysteroid dehydrogenase type 2 (11β-HSD2) activity [9,11]. Unlike steroidal MRAs (e.g., Spironolactone and Eplerenone), which may indirectly influence cortisol-MR interactions via enzymatic pathways, Finerenone directly competes with aldosterone and cortisol for MR binding, thereby inhibiting proinflammatory (e.g., nuclear factor kappa B, NF-κB) and profibrotic (e.g., transforming growth factor beta, TGF-β) signaling cascades [9,14]. This mechanism is distinct from pathological conditions characterized by impaired 11β-HSD2 activity, where cortisol aberrantly activates MR due to insufficient conversion to cortisone [14,16]. By bypassing enzymatic modulation, Finerenone avoids the risk of cortisol-driven MR overactivation, a harmful consequence of 11β-HSD2 inhibition observed in other therapeutic strategies [14].

Finerenone’s unique dibenzothiophene-based structure confers stronger MR affinity and slower dissociation kinetics compared to aldosterone, ensuring prolonged receptor blockade [9]. Critically, its nonsteroidal design eliminates antiandrogenic activity (20-fold weaker than Spironolactone), resolving a key limitation of first-generation MRAs [5,9].

Clinical Validation

The structural and mechanistic advantages of Finerenone translate to improved clinical tolerability. Phase 3 trials demonstrated negligible endocrine side effects (e.g., ≤1% gynecomastia vs. 10%-15% with Spironolactone), validating its minimal off-target interactions with androgen or progesterone receptors [5,11,17]. Furthermore, Finerenone’s balanced tissue distribution, prioritizing cardiac and renal MR over adipose tissue, reduces renal tubular potassium retention, resulting in lower hyperkalemia incidence (14% vs. 19%-24% with Spironolactone) when managed with protocol-driven dose adjustments [7,10,11]. These outcomes confirm its mechanistic promise: selective MR antagonism without enzymatic interference or systemic hormonal toxicity.

Pharmacokinetic Profile

Finerenone exhibits rapid absorption, reaching peak plasma concentrations (Tmax) within two hours, which contributes to its prompt onset of therapeutic action. Its high oral bioavailability (~70%) ensures sufficient systemic exposure to achieve clinical efficacy [9,10]. The prolonged terminal elimination half-life (~20 hours) supports once-daily dosing, enhancing patient adherence and reducing dosing frequency-related errors [11,18]. Finerenone undergoes hepatic phase 1 metabolism via cytosolic enzymes, with subsequent biliary excretion of metabolites, minimizing reliance on renal clearance [9,18]. This pharmacokinetic profile reduces the risk of drug accumulation in patients with renal impairment, a critical advantage for its use in CKD populations [11,18].

Food intake, particularly high-fat meals, delays the absorption rate of finerenone, prolonging the time to reach Tmax by approximately one to two hours. However, total systemic exposure remains unaffected, ensuring no compromise in overall efficacy [11]. This allows flexibility in administration with or without food, though consistent dosing relative to meals is recommended to maintain stable plasma concentrations. Notably, while high-fat meals may transiently delay metabolite clearance, this has no clinically significant impact on therapeutic outcomes [11].

Clinical applications of finerenone

The MR plays a central role in the development and progression of chronic HF, CKD, and proteinuria in primary and secondary glomerular diseases [3]. Additionally, the therapeutic utility of both steroidal and nonsteroidal MRAs is significantly limited by the risk of hyperkalemia, a common and potentially serious adverse effect [6].

As a potential solution, Finerenone, a selective nonsteroidal MRA, has emerged with promising results. Notably, the structure of Finerenone is chemically unrelated to other MRAs, setting it apart. Preclinical and clinical studies have demonstrated its comparatively lower risk of inducing hyperkalemia, a longstanding challenge with traditional MRAs, with trial data showing a 14% incidence vs. 19%-24% with Spironolactone, alongside protocol-driven management to mitigate this risk [9]. Furthermore, Finerenone has been shown to be a highly effective therapeutic option in delaying CKD progression and reducing the risk of kidney failure in patients with T2DM and CKD, as demonstrated in the FIDELIO-DKD trial [11].

Findings from one early-phase clinical study into Finerenone 's effect on CKD indicated significant benefits in systolic and diastolic blood pressure and the left ventricular mass seen in patients with nondiabetic proteinuric kidney disease that had not previously been observed with steroidal MRAs [19]. These remarkable results from the initial study prompted further investigation in advanced clinical trials, which demonstrated renoprotection by Finerenone in patients with T2DM and CKD, heart failure with reduced ejection fraction (HFrEF), and increased CV risk [6,11,20]. These compelling findings strongly suggest that selective, nonsteroidal MRAs could potentially revolutionize treatment in the aforementioned settings, offering myriad advantages, including a notable reduction in the occurrence of hyperkalemia [18].

Treatment of Chronic Kidney Disease

The FIDELIO-DKD trial, a phase 3 randomized, double-blind, placebo-controlled study, evaluated Finerenone in 5,734 patients with CKD and T2DM. Over a median follow-up of 2.6 years, Finerenone significantly reduced the primary composite outcome of kidney failure, sustained ≥40% decline in estimated glomerular filtration rate (eGFR), or renal death by 18% (HR, 0.82; 95% CI, 0.73-0.93; p = 0.001) compared to placebo. Additionally, CV events (death, nonfatal myocardial infarction, nonfatal stroke, or hospitalization for HF) were reduced by 14% (HR, 0.86; 95% CI, 0.75-0.99; p = 0.034), underscoring its cardiorenal benefits [11].

The FIGARO-DKD trial extended these findings to 7,437 patients with earlier stage CKD and T2DM. Finerenone reduced the risk of CV events (death, nonfatal myocardial infarction, nonfatal stroke, or hospitalization for HF) by 14% (HR, 0.87; 95% CI, 0.76-0.98; p = 0.026) and slowed CKD progression, demonstrating efficacy across a broader CKD spectrum [12].

The prespecified Finerenone in Chronic Kidney Disease and Type 2 Diabetes (FIDELITY) pooled analysis (combining FIDELIO-DKD and FIGARO-DKD) included 13,026 patients and confirmed Finerenone’s consistent benefits: a 23% reduction in kidney outcomes (HR, 0.77; 95% CI, 0.67-0.88) and a 14% reduction in CV events (HR, 0.86; 95% CI, 0.78-0.95), irrespective of baseline CKD severity [21]. These trials establish Finerenone as a pivotal therapy for mitigating both renal and CV risks in patients with CKD and T2DM.

Management of HF With Reduced Ejection Fraction

HFrEF is a serious CV disorder with high morbidity and mortality, especially when patients are hospitalized. Though the survival of patients with HFrEF has significantly improved over the last two decades, it remains exceedingly poor and discouraging [1]. Furthermore, the prevailing pandemic of diabetes mellitus and related complications like hypertension and obesity has emphasized the urgent and imperative need for the implementation of effective management strategies in the treatment of HF [2]. In this regard, MRAs have emerged as a promising therapeutic option for this debilitating condition [6].

MRAs primarily function by selectively targeting and neutralizing the MRs that are prominently expressed in crucial CV sites such as the heart, vasculature, and kidneys. By doing so, they successfully attenuate the deleterious effects of fibrosis and inflammation, which are known to exacerbate HF and impede its treatment outcomes [13]. It is important to note that the activation of MR occurs in response to the action of aldosterone, a hormone that is primarily synthesized and secreted from the zona glomerulosa of the adrenal gland [22]. Notably, aldosterone predominantly functions as a sodium-retaining hormone, which, in turn, contributes to the pathophysiology of HF [3]. Accumulating evidence has consistently demonstrated a substantial increase in aldosterone levels among patients afflicted with HF, as well as in experimental rat models [4]. Moreover, it has been ascertained that plasma aldosterone levels are closely associated with adverse health outcomes in individuals suffering from HF [23].

MRAs not only decrease mortality and morbidity in patients with HFrEF but also improve health outcomes in patients with hypertension and CKD [15]. However, hyperkalemia is a major adverse effect of MRA therapy because it decreases renal function and renal excretion of potassium [5]. Despite significant advancements over six decades, the development of novel MRAs has been challenged not only by scientific hurdles, such as optimizing receptor selectivity and minimizing off-target effects, but also by practical barriers, including commercial viability, regulatory complexities, and pharmacokinetic challenges [18,24]. Therefore, there is an urgent need for further research and innovation in this field to discover new therapeutic options that can successfully target and modulate MR, leading to improved outcomes for patients with HFrEF [24]. MRAs have shown significant promise in improving HFrEF patient outcomes [22], but further exploration and development of novel therapies are necessary to address the limitations and adverse effects associated with current treatment options.

Comparative mechanisms and distinction of finerenone among MRAs

Finerenone represents a significant therapeutic advancement as a nonsteroidal, selective MRA, designed to overcome the limitations of traditional steroidal MRAs such as Spironolactone and Eplerenone. Its unique dibenzothiophene-based structure confers high selectivity for MR while exhibiting negligible affinity for androgen or progesterone receptors, thereby minimizing endocrine-related adverse effects like gynecomastia and menstrual irregularities observed with Spironolactone [25]. In contrast, Eplerenone, a second-generation steroidal MRA, retains residual glucocorticoid receptor binding, which limits its safety in renal impairment, while Spironolactone’s moderate receptor selectivity and strong antiandrogenic/progestogenic activity contribute to hormonal toxicity [5,15].

Mechanistically, Finerenone induces distinct conformational changes in MR by binding to a unique hydrophobic pocket, preferentially inhibiting proinflammatory (e.g., NF-κB) and profibrotic (e.g., TGF-β) signaling pathways more effectively than steroidal MRAs [26]. This targeted antagonism contrasts with the broader inhibition of sodium retention and potassium excretion by Spironolactone and Eplerenone, which exacerbates hyperkalemia risk [6,11]. Furthermore, Finerenone’s physicochemical properties, including reduced lipophilicity and balanced tissue distribution, minimize accumulation in adipose tissue and renal tubules, avoiding the systemic toxicity associated with the widespread penetration of steroidal MRAs [9,10].

Clinically, Finerenone demonstrates cardiorenal benefits in patients with T2DM and CKD, a high-risk population with overlapping metabolic and renal dysfunction. In the FIDELIO-DKD trial, Finerenone reduced the composite risk of kidney failure, sustained eGFR decline, or renal death by 18%, alongside a 14% reduction in CV events [11]. The FIGARO-DKD trial further highlighted its CV benefits, showing a 14% risk reduction in CV mortality and morbidity across broader CKD stages [12]. While direct comparisons with steroidal MRAs are limited by differing trial populations, Eplerenone’s 15% mortality reduction was observed in post-myocardial infarction HF (EPHESUS trial), Spironolactone’s 30% mortality benefit was in severe HF (RALES trial), and Finerenone’s improved tolerability in its target population is notable [6,15].

A key advantage of Finerenone lies in its safety profile. Hyperkalemia incidence with Finerenone (14%) is lower than with Spironolactone (19%-24%) and comparable to placebo, attributed to its balanced tissue distribution (preferential targeting of cardiac and renal MR) and reduced lipophilicity, which minimizes renal accumulation [9-11]. While Finerenone exhibits a comparatively favorable safety profile relative to steroidal MRAs, clinically relevant risks such as hyperkalemia (14% incidence in trials) and hypotension necessitate vigilant monitoring and protocol-driven management [7,11]. These risks, though lower than those associated with Spironolactone or Eplerenone, underscore the importance of regular serum potassium checks and dose adjustments in high-risk populations. For example, Spironolactone’s high lipophilicity drives widespread tissue penetration, contributing to off-target hormonal effects (e.g., gynecomastia in 10%-15% of patients) and renal potassium retention. In contrast, Finerenone’s selective distribution mitigates these issues [5,17].

Despite this, hyperkalemia remains the most common reason for discontinuation in Finerenone trials (2.3% in FIDELIO-DKD vs. 0.9% with placebo). Finerenone’s reduced lipophilicity enables proactive management strategies, such as dose reduction (halving the dose for serum potassium >5.5 mmol/L), temporary discontinuation for severe hyperkalemia (>6 mmol/L), dietary modifications, and routine monitoring, to succeed with fewer complications than steroidal MRAs [7,11,27].

Beyond hyperkalemia, Finerenone’s endocrine safety is further evidenced by low rates of gynecomastia (≤1% vs. 10%-15% with Spironolactone), underscoring its potential as a safer long-term therapy in CKD and diabetes populations [5,17]. Tolerability data from trials such as Mineralocorticoid Receptor antagonist Tolerability Study-Heart Failure and FIDELITY underscore Finerenone’s suitability for long-term use. Hyperkalemia occurred more frequently with Finerenone than placebo (14% vs. 9%), but rates of other adverse events, including gastrointestinal disturbances and urinary tract infections, were comparable between the groups [11]. Proactive monitoring and dose adjustments minimized severe hyperkalemia-related discontinuations (2.3% vs. 0.9% placebo), reinforcing its manageable safety profile in high-risk cohorts [7,11]. Table 1 summarizes the pharmacological and clinical distinctions between Finerenone and steroidal MRAs.

**Table 1:** The pharmacological and clinical distinctions between finerenone and steroidal MRAs ^*^Lower risk due to balanced tissue distribution; incidence based on pooled data from FIDELIO-DKD and FIGARO-DKD trials [11,12], risk mitigated by balanced tissue distribution and protocol-driven dose adjustments ^¥^Rates from EPHESUS trial in post-MI HF populations [15], risk persists in advanced CKD due to residual renal accumulation ^#^Higher risk in CKD patients due to lipophilic accumulation and reduced potassium excretion, risk is dose- and CKD-dependent; incidence ranges reflect dose-dependent risk (e.g., 25-50 mg/day) [5,7] ^§^Balanced means preferential targeting of the heart/kidneys ^‡^Widespread means lipophilic-driven penetration into adipose/renal tissue CKD: chronic kidney disease; CV: cardiovascular; FIDELIO-DKD: Finerenone in Reducing Kidney Failure and Disease Progression in Diabetic Kidney Disease; EPHESUS: Eplerenone Post-Acute Myocardial Infarction Heart Failure Efficacy and Survival Study; RALES: Randomized Aldactone Evaluation Study; MRA: mineralocorticoid receptor antagonist; FIGARO-DKD: Finerenone in Reducing Cardiovascular Mortality and Morbidity in Diabetic Kidney Disease; MI: myocardial infarction; HF: heart failure

Feature	Finerenone	Eplerenone	Spironolactone
Generation	Third	Second	First
Structure	Nonsteroidal	Steroidal	Steroidal
Mineralocorticoid receptor selectivity	High	Moderate to high	Moderate
Affinity to sex hormone receptors	Low (minimal off-target)	Low	High (androgen/progesterone)
Hyperkalemia incidence	Lower (14%)^*^	Moderate (15%-18%)^¥^	Higher (19%-24%)^#^
Tissue penetration	Balanced (vascular-focused)^§^	Moderate	Widespread (lipophilic)^‡^
Endocrine side effects	≤1%	<5%	Dose-dependent gynecomastia (10%-15% at >50 mg/day), menstrual irregularities
Diuretic effect	Weak	Weak	Strong (K^+^-sparing)
Key considerations	Preferential renal/heart targeting	Caution in advanced CKD	Dose adjustments in CKD; monitor K^+^ level
Mortality reduction	14% CV events (FIDELIO-DKD)	15% CV death (EPHESUS)	30% all-cause (RALES)
Hospitalization	14% reduction	17% reduction	35% reduction
Renoprotection	18% kidney risk reduction	No significant CKD benefit	Limited data in CKD

Finerenone key clinical trials and studies

Finerenone has been extensively studied in clinical trials across diverse populations with CKD, T2DM, and HF. Below is a chronological overview of key trials, highlighting Finerenone’s therapeutic potential and safety profile. A summary table is provided for quick reference (Table 2).

**Table 2:** Key finerenone clinical trials ACEi: angiotensin-converting enzyme inhibitor; ARB: angiotensin receptor blocker; ARTS-DN: Mineralocorticoid Receptor Antagonist Tolerability Study-Diabetic Nephropathy; CI: confidence interval; CKD: chronic kidney disease; CV: cardiovascular; eGFR: estimated glomerular filtration rate; FIDELIO-DKD: Finerenone in Reducing Kidney Failure and Disease Progression in Diabetic Kidney Disease; FIDELITY: Finerenone in Chronic Kidney Disease and Type 2 Diabetes; FIGARO-DKD: Finerenone in Reducing Cardiovascular Mortality and Morbidity in Diabetic Kidney Disease; FINEARTS-HF: FINerenone trial to investigate Efficacy and sAfety superioR to placebo in paTientS with Heart Failure; HFmrEF/HFpEF: heart failure with mildly reduced/preserved ejection fraction; LVEF: left ventricular ejection fraction; HR: hazard ratio; MRA: mineralocorticoid receptor antagonist; T1DM: type 1 diabetes mellitus; T2DM: type 2 diabetes mellitus; UACR: urinary albumin-to-creatinine ratio; FINE-ONE: FINErenone in participants with type-ONE diabetes and chronic kidney disease

Trial name	Phase	Year	Population	Intervention	Key outcomes	Safety	Significance
ARTS-DN [28] (earlier-stage, dose-finding study)	2b	2014	823 patients, T2DM + persistent albuminuria (UACR ≥30 mg/g)	Finerenone (1.25-20 mg/day) vs. placebo	38% reduction in UACR at highest dose (p < 0.001)	Hyperkalemia is dose-dependent but manageable; low discontinuation rates	First evidence of Finerenone’s renoprotective effects; supported phase 3 trials
FIDELIO-DKD [11] (used time-to-first-event composites)	3	2020	5,734 patients, T2DM + advanced CKD (eGFR 25-60 mL/minute, UACR ≥30 mg/g)	Finerenone vs. placebo (+ ACEi/ARB)	18% reduction in kidney failure/sustained eGFR decline/renal death (HR, 0.82; p = 0.001); 14% reduction in CV events (HR, 0.86; p = 0.034)	Hyperkalemia: 18.3% vs. 9% (placebo); discontinuation: 2.3% vs. 0.9%	Established Finerenone’s dual cardiorenal benefits in advanced CKD
FIGARO-DKD [12] (used time-to-first-event composites)	3	2021	7,437 patients, T2DM + earlier CKD (eGFR ≥25 mL/minute, UACR ≥30 mg/g)	Finerenone vs. placebo (+ACEi/ARB)	Time-to-first-event composites: 13% reduction in CV events (HR, 0.87; p = 0.03); slowed CKD progression (nonsignificant trend)	Hyperkalemia: 10.8% vs. 5.3% (placebo)	Extended Finerenone’s CV benefits to earlier CKD stages
FIDELITY [29]	Pooled	2022	13,026 patients from FIDELIO-DKD + FIGARO-DKD (broad CKD stages)	Pooled analysis	23% reduction in kidney outcomes (HR, 0.77; 95% CI, 0.67-0.88); 14% reduction in CV events (HR, 0.86; 95% CI, 0.78-0.95)	Consistent safety profile across trials	Confirmed Finerenone’s efficacy across CKD severities; dual-organ protection
FINEARTS-HF [27] (used total HF events + CV death composite endpoint)	3	2024	6,215 patients, HFmrEF/HFpEF (LVEF >40%)	Finerenone vs. placebo	12% reduction in total HF events + CV death (HR 0.88; p = 0.02)	Hyperkalemia: 10.5% vs. 4.7% (placebo)	First MRA to show efficacy in HFpEF, a population with limited therapies
FINE-ONE [30]	3	Ongoing	1,650 adults, T1DM + CKD (UACR ≥200 mg/g or eGFR 25-90 mL/minutes)	Finerenone vs. placebo (+ACEi/ARB)	Primary outcome: time to kidney failure/sustained eGFR decline/renal death	Monitoring hyperkalemia and renal safety	Aims to extend Finerenone’s benefits to T1DM, a high-risk, understudied population

ARTS-DN (2014)

The Mineralocorticoid Receptor Antagonist Tolerability Study-Diabetic Nephropathy (ARTS-DN) phase 2b trial evaluated Finerenone’s safety and efficacy in 823 patients with T2DM and albuminuric CKD [28]. Participants received varying Finerenone doses (1.25-20 mg/day) or placebo alongside RAAS inhibitors. Results showed a dose-dependent 38% reduction in albuminuria (urinary albumin-to-creatinine ratio, UACR) at the highest dose (p < 0.001), with manageable hyperkalemia. ARTS-DN provided foundational evidence for Finerenone’s renoprotective potential, paving the way for phase 3 trials.

FIDELIO-DKD (2020)

The FIDELIO-DKD pivotal phase 3 trial enrolled 5,734 patients with T2DM and advanced CKD. Over 2.6 years, Finerenone reduced the primary renal composite outcome (kidney failure, ≥40% eGFR decline, renal death) by 18% (HR, 0.82; p = 0.001) and the CV composite outcome (death, myocardial infarction, stroke, and HF hospitalization) by 14% (HR, 0.86; p = 0.034). Hyperkalemia occurred in 18.3% of Finerenone-treated patients vs. 9% with placebo, but discontinuations were rare (2.3%). Management strategies included protocol-driven dose reductions (from 20 to 10 mg daily) for serum potassium levels >5.5 mmol/L, temporary discontinuation for levels >6.0 mmol/L, and dietary counseling to restrict potassium intake. Regular monitoring (baseline, four weeks, and every four months thereafter) ensured early detection and intervention [7,11]. These measures underscore Finerenone’s manageable safety profile in advanced CKD.

FIGARO-DKD (2021)

In 7,437 patients with T2DM and earlier stage CKD, Finerenone reduced CV events by 14% (HR, 0.87; p = 0.03) over 3.4 years with a trend toward slower CKD progression [12]. Hyperkalemia rates were lower (10.8% vs. 5.3%) than in FIDELIO-DKD, likely due to the inclusion of earlier stage CKD patients. Proactive management included prespecified dose adjustments (e.g., halving the dose for potassium >5.5 mmol/L), monthly serum potassium monitoring during the first year, and patient education on avoiding high-potassium diets and nephrotoxic agents [7,12]. Only 1.2% of participants discontinued treatment due to hyperkalemia, reflecting effective mitigation strategies. While renal benefits were less pronounced than in FIDELIO-DKD, this trial highlighted Finerenone’s broad applicability across CKD stages.

FIDELITY (2022)

Combining 13,026 patients, pooled analysis of FIDELIO-DKD and FIGARO-DKD (FIDELITY) confirmed Finerenone’s 23% reduction in kidney outcomes (HR, 0.77; 95% CI, 0.67-0.88) and 14% reduction in CV events (HR, 0.86; 95% CI, 0.78-0.95) [29]. Benefits were consistent across CKD stages, reinforcing Finerenone’s dual cardiorenal protection in high-risk T2DM populations.

FINEARTS-HF (2024)

FINerenone trial to investigate Efficacy and sAfety superioR to placebo in paTientS with Heart Failure (FINEARTS-HF) phase 3 trial enrolled 6,215 heart failure with mildly reduced/preserved ejection fraction (HFmrEF/HFpEF) patients [27]. Finerenone reduced total HF events and CV death by 12% (HR, 0.88; p = 0.02), offering hope for a population with limited therapeutic options. Hyperkalemia was more frequent in the Finerenone group (10.5%) compared with placebo (4.7%), but severe hyperkalemia (>6 mmol/L) occurred in only 1.8% of patients. Protocolized management included temporary dose interruption, potassium-lowering therapies (e.g., diuretics or sodium polystyrene sulfonate), and intensified monitoring (biweekly during dose titration). No fatalities were attributed to hyperkalemia, demonstrating the feasibility of Finerenone in HFmrEF/HFpEF with vigilant oversight [27,31].

*FINErenone in Participants With Type-ONE Diabetes and Chronic Kidney Disease​​*​​​​​* (Ongoing)*

Finerenone in Adults with CKD and Type 1 Diabetes phase 3 trial (NCT05901831) aims to evaluate Finerenone’s effects in 1,650 type 1 diabetes mellitus (T1DM) patients with CKD [30]. Results will determine if Finerenone’s benefits extend beyond T2DM in this high-risk population.

Future directions, potential areas for research, and new opportunities

For the past four decades, most therapies used to treat HF have targeted neurohormonal feedback loops driven by the sympathetic nervous system and the RAAS. These lifesaving and disease-modifying therapies have proven highly effective [1]. However, despite preclinical success, several agents have failed to demonstrate clinical benefit in trials. These failures are multifactorial, attributable not only to high-risk population inclusion, suboptimal therapy implementation, or interpretive challenges [24], but also to trial design limitations (e.g., inadequate statistical power, heterogeneous cohorts, or poorly defined endpoints), drug-specific issues (e.g., pharmacokinetic variability, off-target effects, or insufficient receptor engagement), and unexpectedly low placebo event rates that obscure treatment effects [1,24,32].

Additionally, MRAs may lose efficacy over time due to specific mechanisms such as MR downregulation, aldosterone breakthrough (persistent aldosterone production despite RAAS inhibition), or progressive cardiorenal disease outpacing receptor blockade [7,18]. Compensatory activation of alternative pathways (e.g., glucocorticoid receptors) and tissue-specific resistance driven by chronic inflammation or fibrosis further contribute to therapeutic limitations [18,26]. Consequently, there remains a critical need to develop novel MRAs with enhanced potency, selectivity, and safety profiles to address these gaps [18].

Finerenone stands out as a unique nonsteroidal and selective MRA, which has shown improved penetrance into the cardiomyocyte due to its balanced tissue distribution and reduced lipophilicity compared to steroidal MRAs, minimizing off-target accumulation in adipose tissue and kidneys [9,10]. Its favorable metabolic profile stems from structural modifications that eliminate antiandrogenic and progestogenic activity, thereby avoiding steroidal side effects such as gynecomastia, impotence, or menstrual irregularities [5,17]. Furthermore, Finerenone’s nonsteroidal backbone enhances metabolic stability, reducing risks of drug-drug interactions and improving tolerability in patients with renal impairment [9,11].

Finerenone’s efficacy in reducing cardiorenal risk has been validated in pivotal phase 3 trials (FIDELIO-DKD, FIGARO-DKD) involving patients with T2DM and CKD. Ongoing phase 3 studies, such as FINErenone in participants with type-ONE diabetes and chronic kidney disease (NCT05901831), aim to extend these findings to type 1 diabetes and nondiabetic CKD populations, ensuring its therapeutic potential is fully explored [17,30].

Emerging interest is growing in combining Finerenone with sodium-glucose cotransporter-2 inhibitors (SGLT2i) or glucagon-like peptide-1 receptor agonists (GLP-1 RAs) to achieve synergistic cardiorenal protection. Preclinical and clinical evidence suggests complementary mechanisms: Finerenone targets MR-driven inflammation and fibrosis, while SGLT2i and GLP-1 RAs improve hemodynamic load, glycemic control, and tubular function [33]. The COmbinatioN effect of FInerenone anD EmpaglifloziN in participants with chronic kidney disease and type 2 diabetes using a UACR Endpoint study trial (NCT05254002) is evaluating the combined effects of Finerenone and empagliflozin on albuminuria reduction in patients with diabetic kidney disease, demonstrating early promise for dual therapy [34]. Similarly, the Combination Efficacy and Safety Study of Finerenone + SGLT2‑Inhibitor in Hospitalized Heart Failure trial will investigate Finerenone alongside SGLT2i in HF patients, aiming to reduce CV mortality and hospitalization rates [35]. A network meta-analysis by Zhang et al. highlighted that while SGLT2i outperforms Finerenone in reducing renal outcomes and HF hospitalizations, their combination could bridge efficacy gaps, particularly in patients with advanced CKD or residual CV risk [33,34]. Future research should prioritize long-term safety and efficacy data for these combinations, explore optimal dosing regimens, and identify biomarkers to guide personalized therapy.

While trials such as FINEARTS-HF demonstrate Finerenone's potential in reducing HF events and CV death in patients with HFmrEF/HFpEF, longer term real-world data are needed to validate its safety and efficacy beyond controlled trial durations. Specifically, future studies should address hyperkalemia management strategies, renal function decline over extended follow-up periods, and mortality outcomes in broader, unselected populations.

## Conclusions

Finerenone’s nonsteroidal structure and selective MR antagonism offer a promising therapeutic alternative with reduced risks of hyperkalemia and endocrine side effects compared to older MRAs. However, its clinical use requires careful monitoring for hyperkalemia and hypotension, particularly in patients with renal impairment or those on concomitant RAAS inhibitors. Robust evidence from pivotal trials-including FIDELIO-DKD, FIGARO-DKD, and FIDELITY-supports Finerenone’s efficacy in reducing renal and CV adverse outcomes in patients with CKD and T2DM. However, broader guideline adoption, cost-effectiveness evaluations, and real-world validation of its long-term benefits and safety will ultimately determine its clinical integration and impact.
